# Service-based analysis of biological pathways

**DOI:** 10.1186/1471-2105-10-S10-S6

**Published:** 2009-10-01

**Authors:** George Zheng, Athman Bouguettaya

**Affiliations:** 1 Virginia Tech, Blacksburg, Virginia USA; 2 CSIRO ICT Centre, Canberra, ACT Australia

**Keywords:** Service Composition, Drug Discovery Process, Pathway Network, Interesting Segment, Entity Instance

## Abstract

**Background:**

Computer-based pathway discovery is concerned with two important objectives: pathway identification and analysis. Conventional mining and modeling approaches aimed at pathway discovery are often effective at achieving either objective, but not both. Such limitations can be effectively tackled leveraging a Web service-based modeling and mining approach.

**Results:**

Inspired by molecular recognitions and drug discovery processes, we developed a Web service mining tool, named PathExplorer, to discover potentially interesting biological pathways linking service models of biological processes. The tool uses an innovative approach to identify useful pathways based on graph-based hints and service-based simulation verifying user's hypotheses.

**Conclusion:**

Web service modeling of biological processes allows the easy access and invocation of these processes on the Web. Web service mining techniques described in this paper enable the discovery of biological pathways linking these process service models. Algorithms presented in this paper for automatically highlighting interesting subgraph within an identified pathway network enable the user to formulate hypothesis, which can be tested out using our simulation algorithm that are also described in this paper.

## Background

Biological pathways are represented as networks of interactions among biological entities such as cell, DNA, RNA and enzyme. The exposure of biological pathways are expected to deepen our understanding of how diseases come about and help expedite drug discovery for treating them. Computer-based pathway study currently relies on two main approaches of entity/process representation: free-text descriptions and computer models. Free-text based approaches used in GenBank [1], DIP [2], KEGG [3, 4], Swiss-Prot [5], and COPE [6] rely on free text annotations and narratives [7, 8] to target towards human comprehension. One major disadvantage with these approaches is their inherent lack of support for computer-based simulation of these processes. Computer models (e.g., [9–14]) of biological processes, on the other hand, while enabling computer-based simulations of biological processes, are often constructed in isolated environments, limited to the study of known pathways, and lack the ability to facilitate the discovery of new pathways. We propose to use Web service modeling strategy [15] to bridge the gaps between the two representation approaches. Using this strategy, biological processes are modeled as Web service operations, which can be first described and published by one organization, and later *discovered* and *invoked* by *independently* developed applications from other organizations. A service operation may consume some input substance meeting a set of preconditions and then produce some output substance as a result of its invocation. Some of these input and output substances may themselves carry processes that are known to us and thus can be also modeled and deployed as Web services. Domain ontologies containing definition of various entity types would be used by these Web services for describing their operation inputs and outputs. This service oriented process modeling and deployment strategy not only allows for the identification of pathways linking processes of biological entities, as do existing natural language processing approaches (e.g., [16, 17]), but would more importantly bring about unprecedented opportunities for analyzing such pathways right on the Web through direct invocation of involved services. When enough details are captured in these process models, this in-place invocation capability presents an inexpensive and accessible alternative to existing *in vitro* and/or *in vivo* exploratory mechanisms.

The second key contribution of our work is the development of our service mining tool, named PathExplorer, used to discover potentially interesting biological pathways (i.e., composition networks) linking service models of biological processes. Unlike traditional top down service composition approaches that are driven by specific user goals, Web service mining, which aims at the discovery of *any* interesting and useful compositions of Web services, is carried out in a bottom up fashion with no such goals to guide the search process. As a result, it faces the challenge of combinatorial explosion as the number of service models increases. In search for efficient mining algorithms and framework, we drew inspirations from molecular recognitions and drug discovery methodologies and developed several key mining algorithms with performance that is linear to the number of service models that are involved [18].

In [19], we applied our Web service mining framework [20] to service models of biological processes that are deployed using Web Service Modeling eXecution environment (WSMX) [21] for the discovery of biological pathways. These service models are expressed using both Web Service Markup Language (WSML) and Web Services Description Language (WSDL). We then explored the opportunity of evaluating such pathways on the Web through direct invocation of involved services. In [22], we extended our approach to also provide graph-based hints on discovered pathways to help user formulate hypotheses, which can then be either confirmed or rejected based on simulation results, leading to the identification of useful pathways. In this paper, we establish the analogies between molecules and Web services, paving the way for future interdisciplinary exploration of these two seemingly unrelated subjects. We also describe in detail our graph expansion algorithms that are not covered in [22]. The algorithms are used to identify subgraphs linking interesting edges and user selected nodes within an existing pathway network. These subgraphs provide the basis for hypothesis formulation and simulation based evaluation.

## Methods

The bottom up Web service mining inevitably exposes itself to the problem of combinatorial explosion, which, if left unaddressed, renders the mining process unscalable as the number of services involved increases. Nature, however, has provided us with ample examples on how composition takes place in a bottom up fashion. In this section, we first establish analogies between molecular world and Web services world. We then draw inspirations from molecular recognitions and drug discovery processes and present our Web service recognitions mechanisms and mining framework.

### Analogies between molecules and Web services

**Figure 1:**
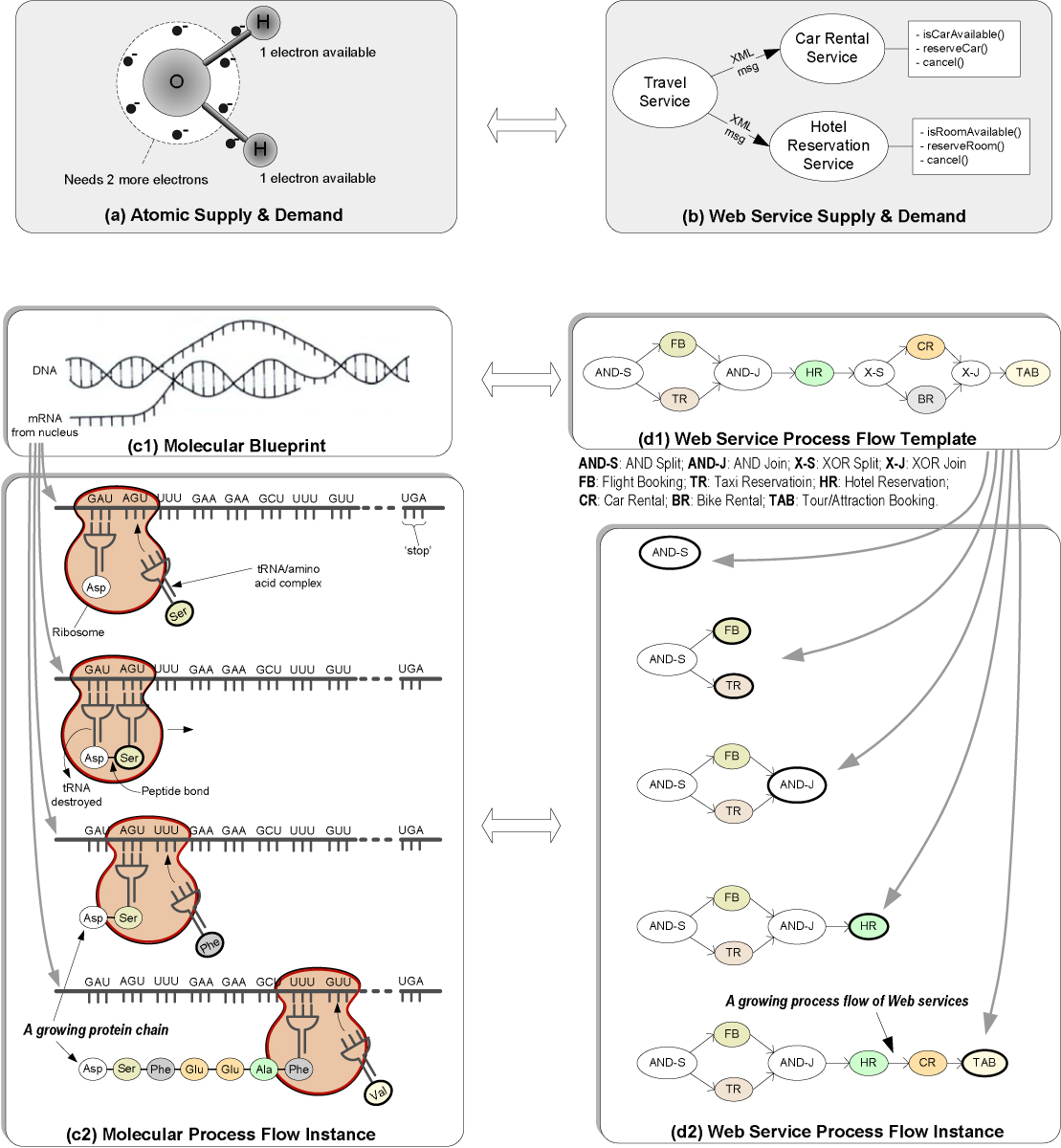
**Analogies between Molecules and Web Services**. (a)An oxygen atom has six electrons in its outer shell and needs twomore electrons to fill the shell. Meanwhile, a hydrogen atom has one electron in its outer shell that can be shared with the oxygen atom. Under the right condition, the oxygen atom shares one of its six electrons with each of the two hydrogen atoms. This form of bonding (called *covalent* bonding) is what holds a water molecule together. (b) The composite travel service needs to know whether a rental car is available within a given time period in a certain location. In addition, it also needs to know whether there is a room vacancy at the same proximity. These inquiries can be fulfilled by invoking operations provided by both the car rental and hotel services. (c) A gene-bearing portion of the DNA double helix is unraveled and information contained in one of the two single strands is transcribed into a *messenger* RNA (mRNA) through molecular recognition (c1). The mRNA is then detached from the DNA strand and serves as a template in the protein building process (c2). This process involves a *transfer* RNA (tRNA), which collects amino acids required to build the protein and carries them to the mRNA. Proteins are assembled on the mRNA one amino acid at a time. Links between adjacent amino acids called *peptide bonds* are established as the protein chain grows. (d) A travel process flow template can be first constructed specifying the logic order of sequential, concurrent (i.e., AND Split *AND-S* and AND Join *AND-J*) and alternative (i.e., OR Split *OR-S* and OR Join *OR-J*) service invocations (d1). Upon receiving a request, a process flow for a composite travel service is instantiated and grows (d2) based on this template.

Web services share similarities in many ways with atoms and molecules in the natural world. At the most basic level, Web services are analogous to atoms, as illustrated in Figures 1(a) and 1(b). In the chemical world, oxygen and hydrogen atoms are two of the most basic building blocks in nature. Under the right condition, the *supply-demand* relationship between the two types of atoms drives them to form *bonds* and, ultimately, a water molecule. Similar supply-demand relationship is also what drives Web services together. Like a molecule that is composed of atoms or simpler molecules, a composite Web service is composed of simpler component Web services. Operation invocations among services are realized through the exchange of eXtensible Markup Language (XML) messages. Similar to electrons in the natural world, these messages bind component Web services into composite services. If we imagine each message as an electron, then this type of message exchange can help establish bonds between Web services. Here, we use bond as a notional concept to indicate the composability between two Web services.

The analogy between the molecular and Web service worlds continues at a more complex process level. In the chemical world, the DNA inside our cells provides a complete genetic blueprint that carries the information required to manufacture the enzyme proteins, which in turn are responsible for orchestrating our body's chemistry. The progression from DNA to mRNA to protein involves a molecular assembly line [23] that follows a remarkable process (Figure 1(c)). Likewise, Web service composition can also involve a complex process (Figure 1(d)) using process template as blueprint for process flow instances. The process template is analogous to the blueprint carried by the mRNA and the process flow instance is analogous to the protein chain.

The similarities between Web services and *molecules* offer some interesting insights. They suggest that like molecules that compose from bottom up as if they are living beings, Web service can also be treated as living beings that *recognize* each other under the right conditions. The process analogy indicates that recognition-triggered service composition may extend to a flow network. Such a flow network can be either designed from top down or emerged from bottom up. As a result, instead of having to search for interesting and useful Web service compositions and composition networks exhaustively, the compositions and composition networks could form "naturally" from bottom up, similar to what is happening in the natural world.

### Web service/operation recognitions

Similar to the molecular world, the natural formation of service compositions is based on automatic recognitions among Web services and their corresponding operations. We have identified the following three service/operation recognition mechanisms that are applicable to Web service models of biological processes:

#### Promotion

**Figure 2:**
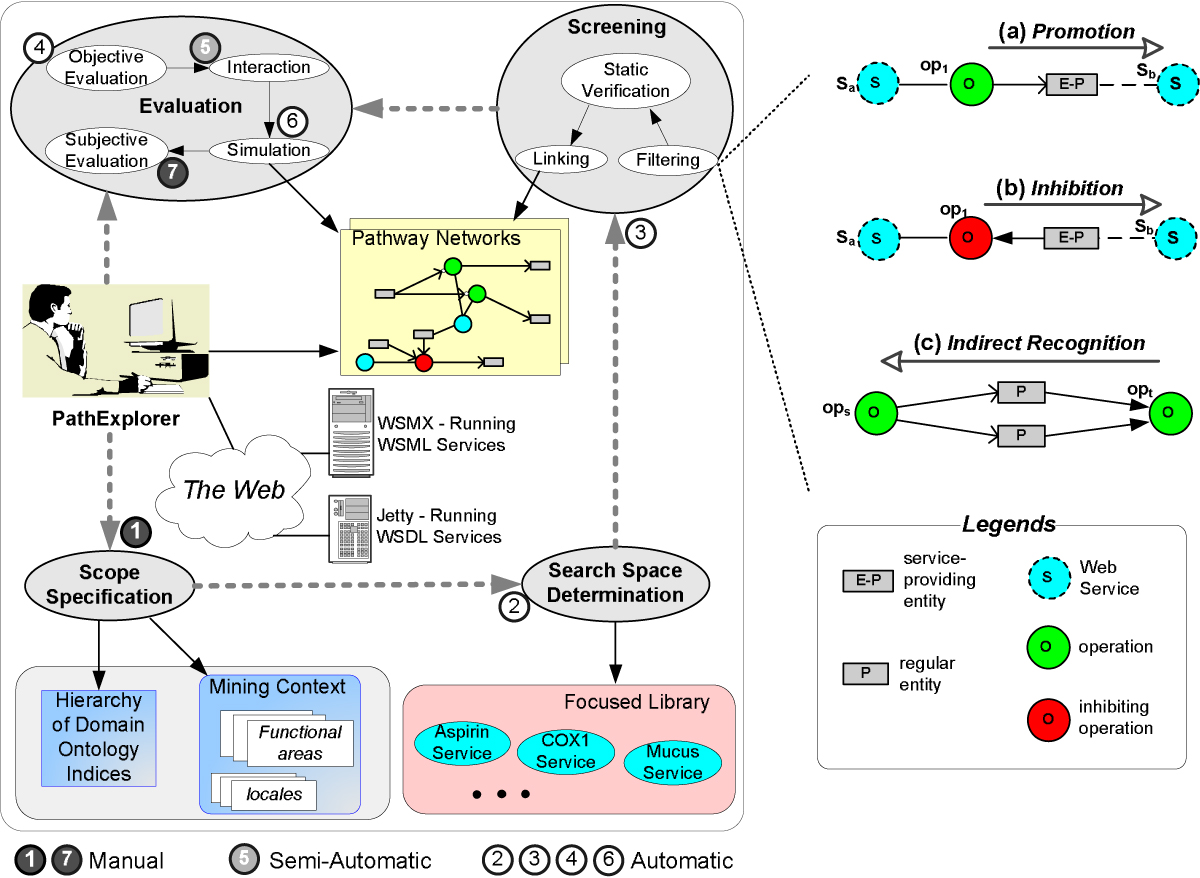
**PathExplorer Architecture**. The left side shows the architecture of PathExplorer. The right side shows the three service/operation recognition patterns that are used by the filtering algorithms in the screening phase.

When operation *op*_1_ of service *s*_*a*_produces an entity (i.e., output parameter) that in turn provides service *s*_*b*_, we say that *s*_*a*_: *op*_1_ promotes *s*_*b*_, as shown in Figure 2(a).

#### Inhibition

When operation *op*_1_ of service *s*_*a*_consumes an entity (i.e., input parameter) that in turn provides service *s*_*b*_, we say that *s*_*a*_: *op*_1_ inhibits *s*_*b*_as shown in Figure 2(b).

#### Indirect Recognition

A *target* operation *op*_*t*_indirectly recognizes a source operation *op*_*s*_, if *op*_*s*_generates some or all input parameters of *op*_*t*_, as shown in Figure 2(c). Indirect recognition is in contrast to the concept of *direct recognition* [20], where an operation can be directly invoked by another. Direct recognition is applicable to fields such as e-commerce but not pathway discovery and is thus not included here. These recognition mechanisms form the basis of the filtering algorithms [20] in our mining framework.

### PathExplorer architecture

As we look for appropriate Web service mining framework, the identification of the similarities between Web services and molecules leads us to the relevance of drug discovery methodologies used in the pharmaceutical industry. A typical drug discovery process [24] involves seven steps:

1. Disease selection,
2. Target hypothesis,
3. Lead compound identification and screening,
4. Lead optimization,
5. Pre-clinical trial,
6. Clinical trial, and
7. Pharmacogenomic optimization.

There are several interesting observations about the process described above. First, the drug discovery process has adopted the strategy of screening molecules (step 3) using "coarse-grained" filtering approach to quickly reduce the search space from the focused library of potential ligands to one that contains those most likely to bind to a protein target with high affinity. It then increases the computation complexity with better accuracy on a reduced search space for lead optimization (step 4). With a much smaller remaining space, the discovery process finally conducts more expensive clinical study for drug evaluation. This is a powerful strategy and can also apply well in the field of Web service mining.

Web service screening could take advantage of some "mining context" to scope down the searching space and identify potentially composable Web services in an early stage. The identification of the composability can be achieved using a "coarse-grained" ontology-based filtering mechanism. Automatic verification and objective analysis can be applied next in a reduced pool of candidate services. A more elaborate runtime simulation mechanism can then be applied towards composed Web service leads in a much smaller search space to investigate the relationships among various composition leads involved in the composition network. Finally, expensive subjective usefulness analysis involving human in the loop can be conducted in an even smaller search space to distinguish those that are truly useful.

Figure 2[18] shows the architecture of PathExplorer, which starts with *scope specification*, a manual phase involving a domain expert defining mining context including functional areas (e.g., cell enzyme, drug functions) and/or locales (e.g., heart, brain) where these functions reside. Based on such mining context, PathExplorer establishes a hierarchy of domain ontology indices to speed up later phases in the mining process. Scope specification is followed by several automatic phases. The first of these is *search space determination*, where the mining context is used to define a focused library of existing Web services as the initial pool for further mining. The next is the *screening* phase, where Web services in the focused library would go through filtering algorithms for the purpose of identifying potentially interesting leads of service compositions or pathway segments. The filtering algorithms are based on the three service/operation recognition mechanisms described earlier.

Based on these recognition mechanisms coupled with a publication/subscription-based algorithm [20], linkages between Web services and their operations in the focused library can be quickly established from bottom-up. These pathway segment leads are then semantically verified based on a subset of operation pre-and post-conditions involving binary variables (e.g., whether the input to an operation is activated) and enumerated properties (e.g., the locale of an operation input). Finally, verified pathway segment leads are linked together using our link algorithms for establishing more comprehensive pathway network.

Discovered pathways from the screening phase are input to the *evaluation* phase, which consists of four sub-phases. *Objective evaluation* identifies and highlights interesting segments of a pathway by checking whether such linkages are novel (i.e., previously unknown). An *interactive* session follows next with the user taking hints from these highlighted interesting segments within the pathway network and picking a handful of nodes representing services, operations and parameters to pursue further. PathExplorer then attempts to link these nodes into a connected graph using a subset of nodes and edges in the original graph. This subgraph provides the user the basis to formulate hypotheses. As an example, such a hypothesis may state that an increase in the dosage amount of Aspirin will lead to the relief of pain, but may inadvertently increase the risk of ulcer in the stomach. These hypotheses can be tested out via *simulation*, which involves PathExplorer invoking the relevant service operations, changing the quantity/attribute value of various entities involved. Simulation results showing the dynamic relationships between these biological entities are then presented to the user, whose *subjective evaluation* finally determines whether the pathway in pursuit is actually useful.

### Service-based modeling of biological processes

**Figure 3:**
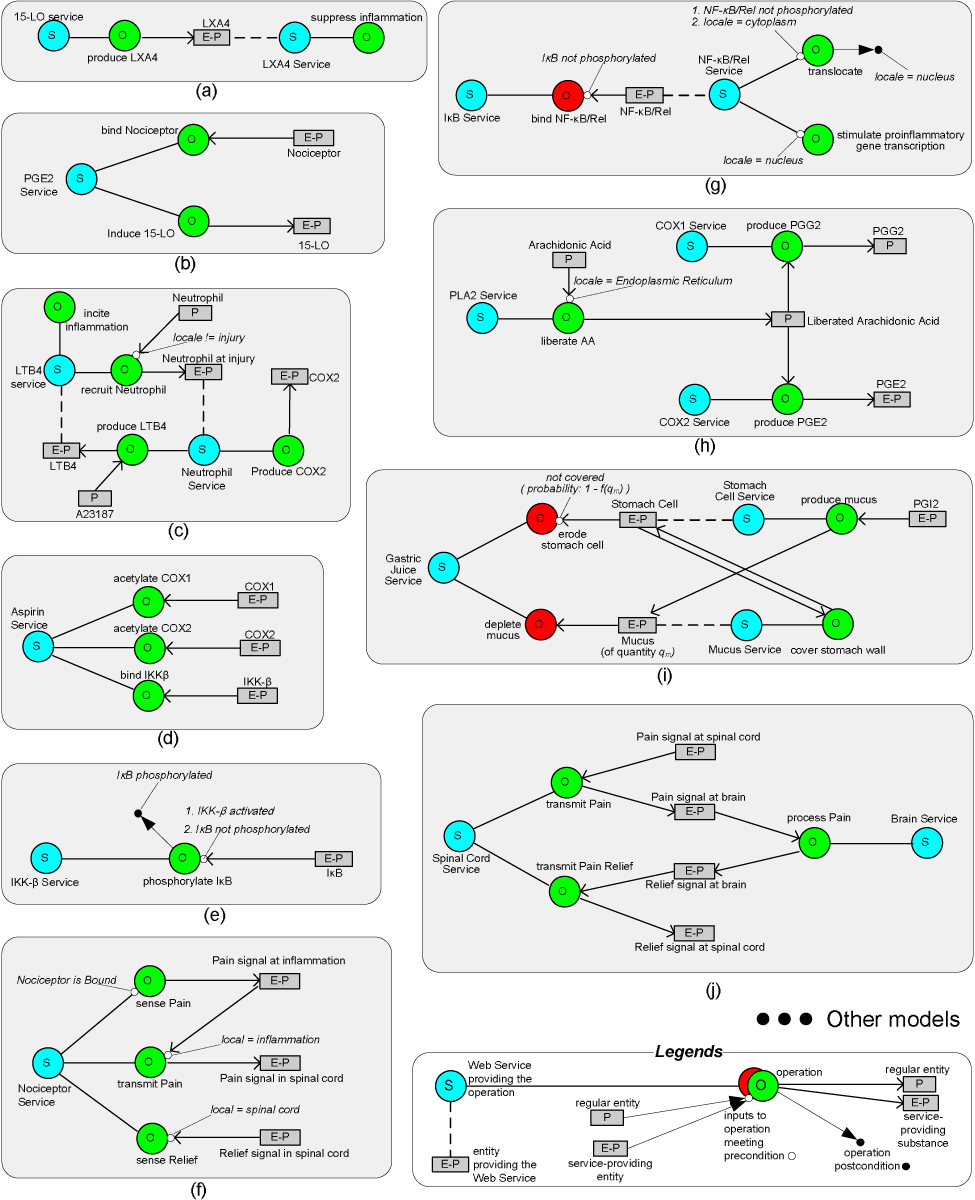
**Examples of Conceptual Process Model and Simple Pathway**. Multiple examples of *promotion*, *inhibition* and *indirect recognition* can be found in these pathways. For example, (a) shows that 15 LO provides an operation called *produce LXA4*, which promotes the service of LXA4. (c) shows that upon injury, LTB4 recruits Neutrophil, promoting its service of producing COX2. (i) shows that Gastric Juice's service can inhibit the services of both Stomach Cell and Mucus. Examples of *indirect recognition* can be found in (h), where PLA2's service can liberate Arachidonic Acid, which can in turn be used as input to either the *produce PGG2* operation of COX1's service or the *produce PGE2* operation of the COX2 service. Examples of pre- and post-conditions can be found in (g), where NF-*κ* B/Rel when not phosphorylated can translocate from cytoplasm to cell nucleus, where it can stimulate proinflammatory gene transcription. NF-*κ* B/Rel's service, however, may be inhibited by the I*κ* B service if NF-*κ* B/Rel is bound by its corresponding operation when I*κ* B is not phosphorylated.

To model biological processes as Web services, we first compiled a list of conceptual process models shown in Figure 3 that are based on [25–27] and [28]. In addition to describing process models, these sources also reveal some simple relevant pathways that can be manually put together. We use process models such as these as references when we develop real Web services. We also use simple pathways manually constructed here as references when we check the correctness of pathways automatically discovered using our mining algorithms.

**Figure 4:**
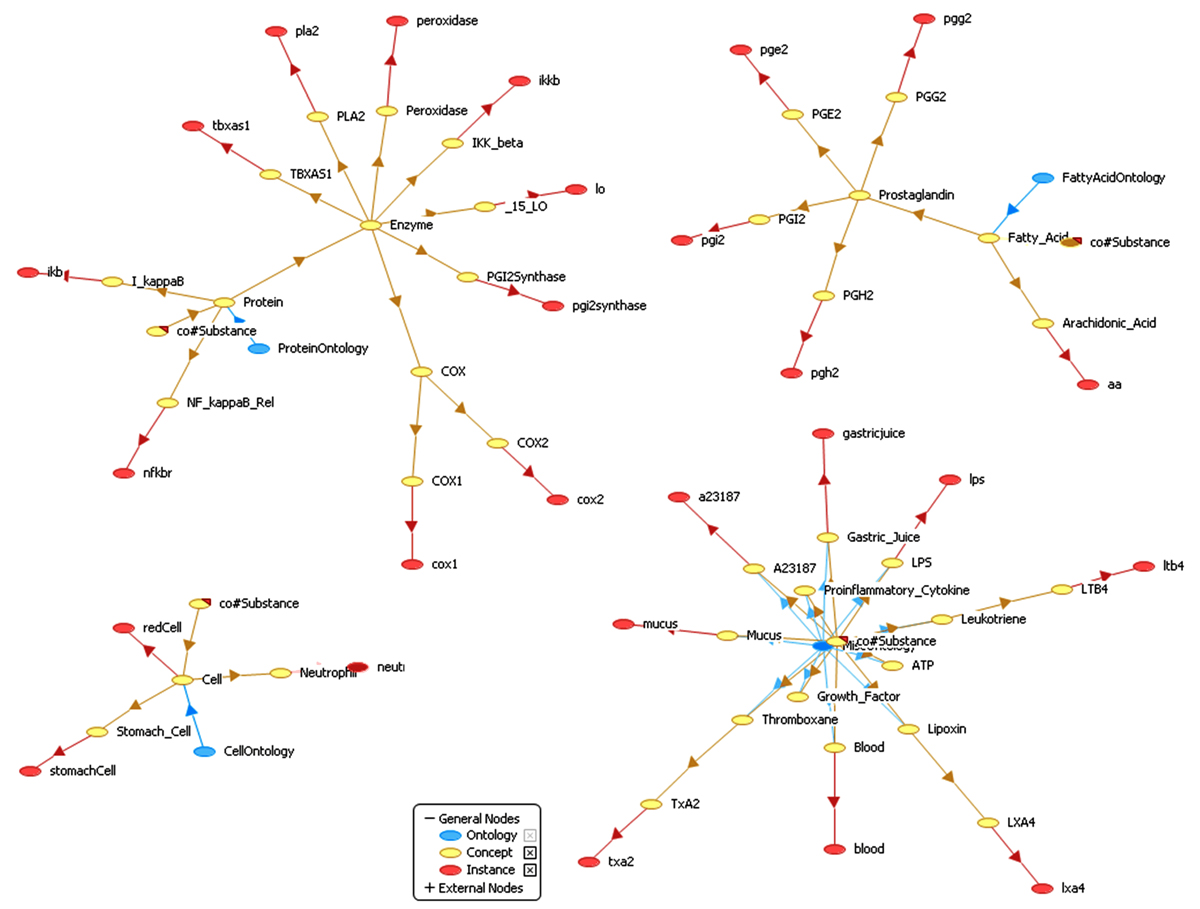
**Example Ontologies Rendered in WSMT**. These ontologies are used in WSML descriptions of Web services for specifying the types of service providing entities and operation input and output substances.

Each of the conceptual process models is next captured in a Java class and exposed through Axis2 [29] running inside a Jetty Web server [30] as a WSDL [31] service. Although the internal details of biological processes can be modeled as WSDL Web services, WSDL itself does not provide elaborate mechanism for expressing the pre- and post-conditions of service operations. WSDL also lacks the semantics needed to unambiguously describe data types used by operation input and output messages. We choose WSML [32] among others (e.g., Web Ontology Language based Web service ontology (OWL-S) [33], WSDL with Semantics (WSDL-S) [34]) to fill this gap due to the availability of WSMX [21], which supports the deployment of ontologies and Web services described in WSML. We categorize biological entities within our mining context into several ontologies. These include *Fatty Acid, Protein, Cell*, and *Drug*. They would all refer to a *Common* ontology containing generic entity types such as *Substance*, the root concept of all entity types. We use *UnknownSubstance* as a placeholder for process inputs that are not fully described in the literature. We also create a *Miscellaneous* ontology capturing definitions of entity types found in the literature that don't seem to belong to any domain. Figure 4 shows several ontologies including those for cells, proteins, fatty acids and the miscellaneous entities rendered in Web Service Modeling Toolkit (WSMT) [35].

**Figure 5:**
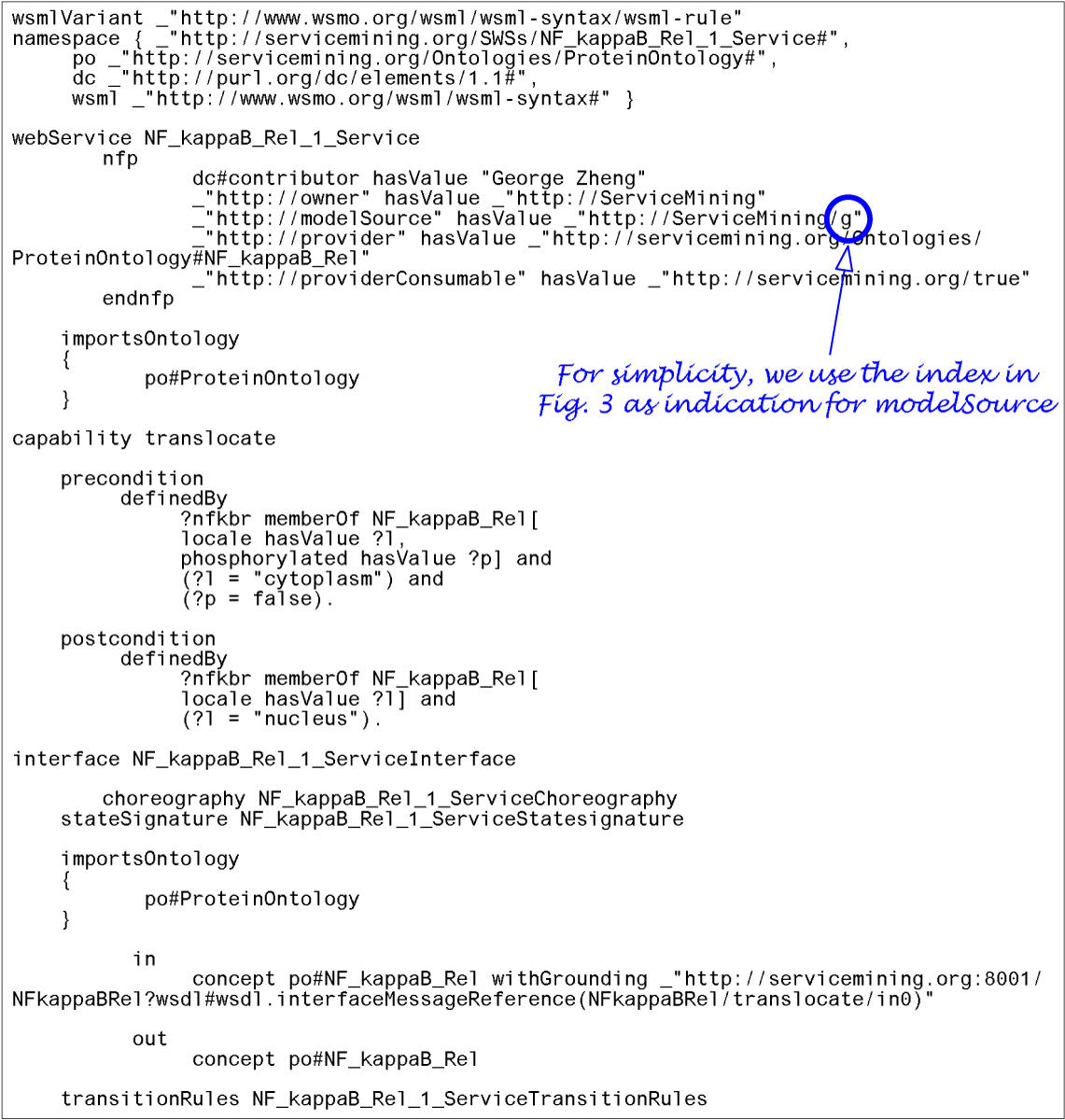
**Semantic Interface Description in WSML**. Example WSML service for *NF_kappaB_Rel*: The capability section states for the precondition that the input entity instance named *nfkbr* should be of type *NF_kappaB_Rel* (defined in the protein ontology). In addition, *nfkbr*'s locale should be cytoplasm and it should not be phosphorylated. The interface section states that input entity *NF_kappaB_Rel* has grounding with the *translocate* operation of the corresponding WSDL service. The output from this service operation should be mapped to *NF_kappaB_Rel* as defined in the protein ontology.

Using these ontologies, we then wrap the semantic interfaces of existing WSDL services as WSML services. WSML supports the descriptions of pre- and post-conditions in the capability section and the ontological type description in the interface section. Figure 5 gives an example of each for the *NF_kappaB_Rel* service. To work with WSML, we have made slight adaptations to our screening algorithms so they can be applied directly to WSML services. First, we add a *provider* property in the non functional properties (nfp) section of each WSML service to indicate the corresponding ontological type of an entity that can provide the service. PathExplorer uses this information to establish the relationship between a service providing entity and the service it provides. Second, we add a *modelSource* property in the nfp section to indicate the source information that the model is based on. Third, we add a *providerConsumable* property in the nfp section to indicate to PathExplorer whether the service providing entity should be consumed along the invocation of its operation. For example, in order for mucus (Figure 3(i)) to cover the wall of stomach, the mucus itself will have to be consumed. Finally, our validation algorithm has been customized to work with the service interrogation APIs of the WSMX runtime library for determining the overlap between the postcondition of a source operation and the precondition of a target operation. Unfortunately, WSML allows for the specification of pre- and post-conditions for only an entire service, but not its individual operations. Thus we have to split services that each originally has multiple operations into several services (e.g., *NF_kappaB_Rel_1_Service* and *NF_kappaB_Rel_2_Service*) so that different conditions can be individually specified for these operations. PathExplorer uses the name of these services to keep track of their relationship and uses that information to merge these services towards the end of the screening phase. During simulation, PathExplorer uses lowering/lifting adapters [19] to convert ontological entity instances used by WSML services to/from Simply Object Access Protocol (SOAP) messages used by WSDL services.

### Pathway visualization and establishment of interesting subgraphs

**Figure 6:**
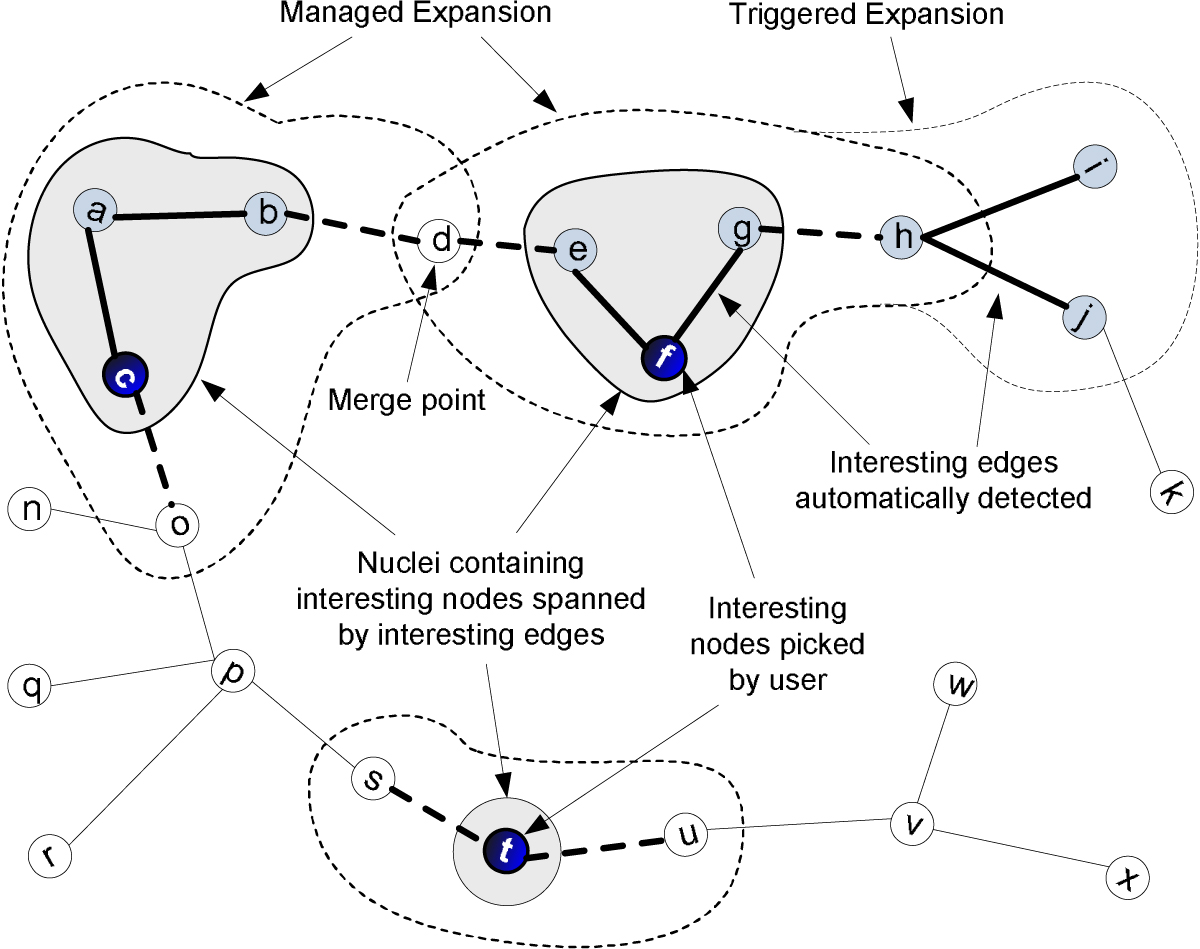
**Expansion of Interesting Segments in Composition Graph**. The expansion of interesting segments involves three steps: 1. Coalescing nodes (e.g., *a, b, c*) linked by interesting edges into a group, 2. Converting interesting nodes (e.g., *t* picked by user) and groups encompassing interesting nodes (e.g., *c, f*) into nuclei, i.e., graph expansion focus nodes, and 3. Incrementally expanding all the nuclei. We use the heuristics of connecting all the interesting nodes using as many interesting edges as possible. To achieve this, whenever a newly encountered node is part of a non-nucleus group (e.g., one that contains *h, i* and *j*), an additional expansion is also triggered and the whole group are engulfed. The expansion stops when all nuclei are connected or when all nodes in the graph are visited.

To support pathway visualization, PathExplorer generates a Graph Markup Language (GraphML) file for each discovered pathway network and uses yEd [36] to render the corresponding graph. We have developed algorithms in PathExplorer to help user, during the evaluation phase, formulate hypotheses that would lead to the identification of useful pathways extended from interesting segments. The addition of the *modelSource* property (Figure 5) in the nfp section allows PathExplorer to identify novel (i.e., interesting) linkages between service models in a discovered pathway network by comparing the source indicator of linkages in the pathway graph representing the three types of service/operation recognitions as shown in Figure 2. PathExplorer then automatically highlights these edges in the graph, presenting them as visual aid to the user for focusing more on nodes that may lead to the identification of useful pathways. After the user selects nodes of interest, PathExplorer attempts to link them into a connected graph to the extent possible using steps illustrated in Figure 6.

**Figure 7:**
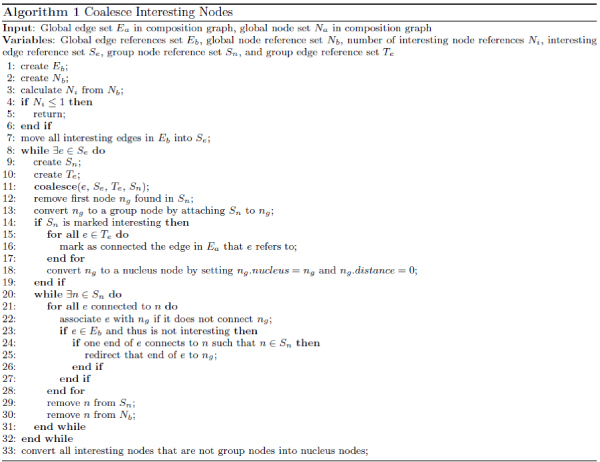
**Algorithm covering steps 1 and 2 in Figure 6 for coalescing interesting nodes**.

**Figure 8:**
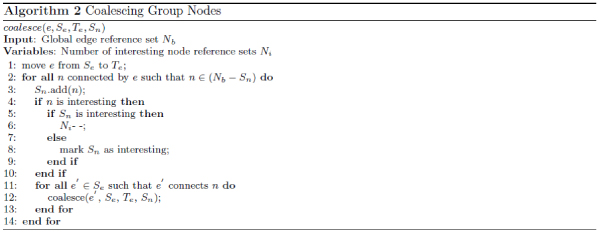
**Algorithm for coalescing group nodes**. This is invoked at line 11 in Figure 7.

Connected graphs identified using the above process are then presented to the user as basis for hypothesis formulation. We list Algorithm 1 used to achieve steps 1 and 2 in Figure 7. We first construct two global reference sets: *E*_*b*_for edges and *N*_*b*_for nodes (lines 1 and 2). Since we are trying to connect all interesting nodes, the algorithm stops when *N*_*i*_≤ 1 (lines 4 to 6). The rest of the algorithm aims at coalescing each group of nodes in *N*_*b*_linked by interesting edges into one node. We first construct *S*_*e*_(line 7) to initially contain all the interesting edge references. For each remaining edge *e* picked from *S*_*e*_(line 8), we construct a group node reference set *S*_*n*_(line 9) and group edge reference set *T*_*e*_Te (line 10). A *coalesce*( ) function (Algorithm 2 as shown in Figure 8) is then invoked to coalesce nodes that *e* connects. Using *coalesce*( ), we first move *e* from the interesting edge reference set *S*_*e*_to the corresponding group edge reference set *T*_*e*_(line 1). Then for each node n, which is in *N*_*b*_but not *S*_*n*_and which *e* connects to (line 2), we add it into the corresponding group node reference set *S*_*n*_(line 3). If *n* is an interesting node (line 4) and *S*_*n*_is already marked as interesting (line 5), then we know that *n* is not the first interesting node in *S*_*n*_, thus we can reduce the number *N*_*i*_of interesting node reference sets by 1 (line 6). If *S*_*n*_is not yet marked as interesting, we need to simply do so (line 8). We then recursively invoke Algorithm 2 in Figure 8 for all other edges in *S*_*e*_that are connected to *n* (lines 11 to 13). It is conceivable that *S*_*e*_, *T*_*e*_and *S*_*n*_may all change as a result of this coalescence process. Going back to Algorithm 1 in Figure 7, code in lines 12 through 31 aims at picking a node from each group as the proxy for the whole group during the incremental expansion phase (step 3). To achieve this, we pick out the first node *n*_*g*_found in *S*_*n*_(line 12) and convert it to a group node (line 13). Lines 14 through 19 converts *n*_*g*_to a nucleus node and marks corresponding edges in the global edge set *E*_*a*_as already connected. Lines 21 through 31 makes *n*_*g*_a surrogate node for all the other nodes in the same group. In line 33, we also convert interesting nodes (e.g., *t* in Figure 6) that are not group nodes into nucleus nodes.

**Figure 9:**
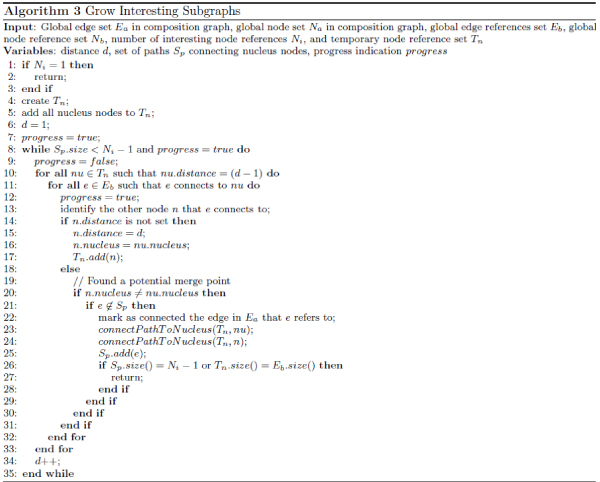
**Algorithm covering step 3 in Figure 6 for growing interesting subgraphs**.

**Figure 10:**
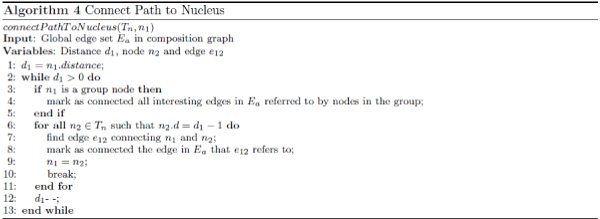
**Algorithm for connecting path to nucleus**. This is invoked at lines 23 and 24 in Figure 9.

Managed expansion described in step 3 is achieved via Algorithm 3 as shown in Figure 9. The algorithm first checks whether there is only one interesting node and it should simply stop (lines 1 to 3). If there are more than one interesting node, it constructs *T*_*n*_, used to keep track of all visited nodes, to initially contain all nucleus nodes (lines 4 and 5). The rest of the algorithm then incrementally expands all the nuclei until they are all connected (line 8 as *N*_*i*_- 1 edges are needed to connect *N*_*i*_nuclei) or when all nodes in the graph have been visited (line 26). In addition, we use variable *progress* (lines 7, 9 and 12) to keep track of the progress of graph expansion and stops algorithm if no progress has been made during the last iteration (line 8). A distance variable *d* is used to manage the incremental expansion. As the expansion progresses, each of the encountered nodes is checked to see whether its distance attribute is already set. If this is not set (line 14), then the node must be a newly encountered node and the algorithm sets the distance (line 15), tags it as belonging to the same nucleus group (line 16) and indicates that the node has been visited by adding it into *T*_*n*_(line 17). If the node is a previously visited node (line 18) and it is associated to a nucleus group different from the current one (line 20), then a merge point (see Figure 6) is potentially encountered. To be sure, the algorithm checks whether the edge extending to the node just encountered has already been visited (line 21). If not, it marks the corresponding edge in *E*_*a*_as connected and then invokes *connectPathToNucleus*( ), indicates that the edge has been visited by adding it into *S*_*p*_(line 25), and checks whether the stop criteria (line 26) have been met. Algorithm 4 in Figure 10 lists the algorithm used in *connectP athT oNucleus*( ) to mark all edges from the encountered node and leading to the corresponding nucleus node as connected. The traversal of a group node (line 3) would trigger additional expansion (line 4) that would mark all interesting edges in the corresponding group also as connected.

### Pathway simulation

**Figure 11:**
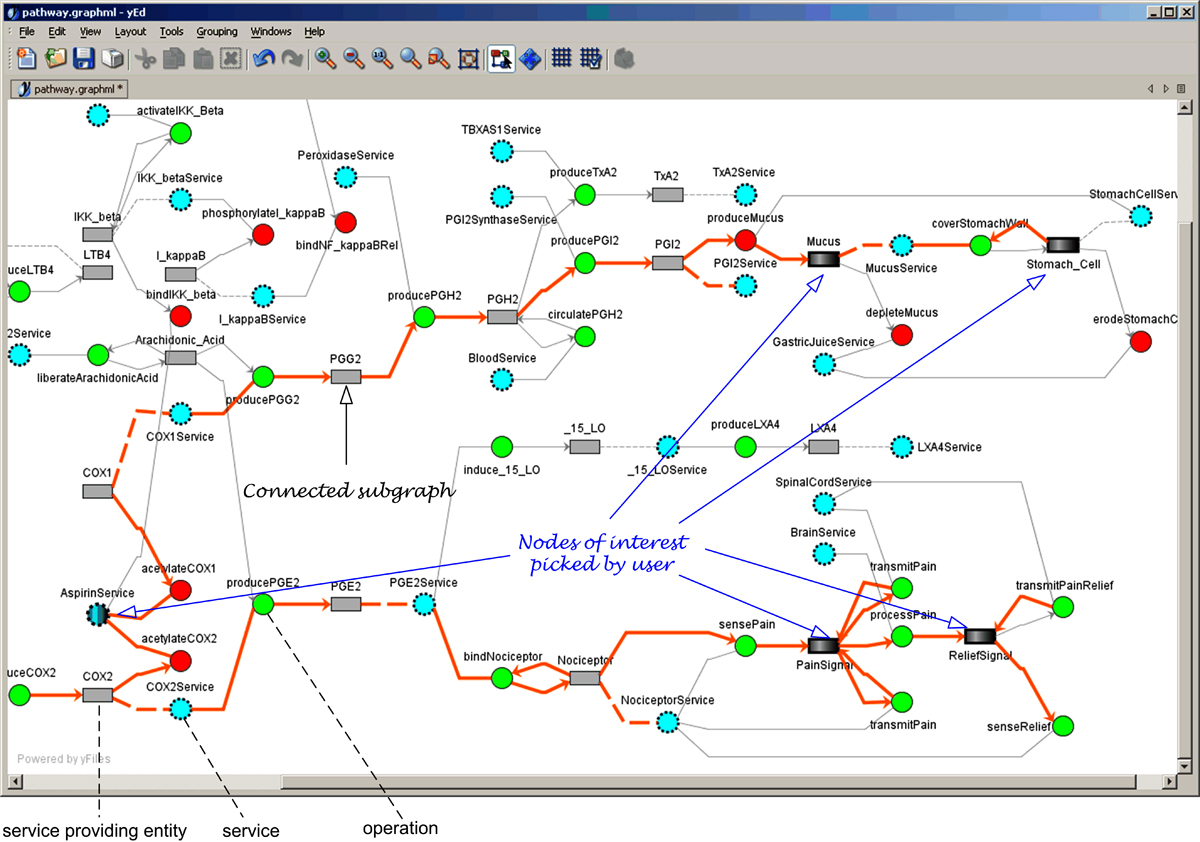
**Discovered Pathway Rendered in yEd**. For brevity, we display only shortened names for nodes in the graph. We keep the full name containing either the ontological path for entity nodes or the WSML service path for both service and operation nodes in a separate description field (not shown here). In addition, we omit in this Figure pre- and post-condition details of operation linking edges such as the two forming a loop between operation *coverStomachWall* and entity *Stomach_Cell*. Such details are kept by our algorithm and used during simulation. The precondition along the upper edge states that Stomach_Cell is not covered by Mucus and the postcondition along the lower edge states that Stomach Cell is covered by Mucus.

Discovered pathway networks are first presented to the user in yEd graphs with interesting linkages highlighted. Once interesting nodes are picked by the user, PathExplorer attempts to use the above process to link them into a connected subgraph, an example of which is shown in Figure 11 and highlighted with thick edges. Such graphs are then presented to the user as basis for hypothesis formulation. We keep track of the pre- and post-condition details of operation linking edges (not shown in Figure 11) in our algorithm as such information along with the ontological entity paths and WSML service paths are needed when we try to invoke these services during simulation. To ensure the correctness of our algorithms, we compared segments within the automatically discovered pathway network with those constructed manually in Figure 3 and found them to be consistent in all cases.

When an operation is to be invoked, the algorithm checks two factors. First, it examines whether all the pre-conditions of the operation are met. An operation that does not have available input entities meeting its preconditions should simply not be invoked. Second, it determines how many instances are available for providing the corresponding service. This factor is needed due to the fact that biological entities of the same type *each* has a discrete service process that deals with input and output of a finite proportion. The available instances of a particular service providing entity will drive the amount of various other entities they may consume and/or produce. For this reason, the algorithm treats each entity node in a pathway network such as one shown in Figure 11 as a container of entity instances of the noted ontology type. In some cases, the service provider is also used as an input parameter. For example, the *sensePain* operation from the *NociceptorService* in Figure 3(f) has a precondition stating that the *Nociceptor* itself should be bound in order to provide this service. In order to express this precondition, we decided to include the service providing entity also as an input parameter. In cases such as this, the number of service providing instances will be determined by checking further whether each of the service providing entity instances also meets the precondition of the corresponding operation.

**Figure 12:**
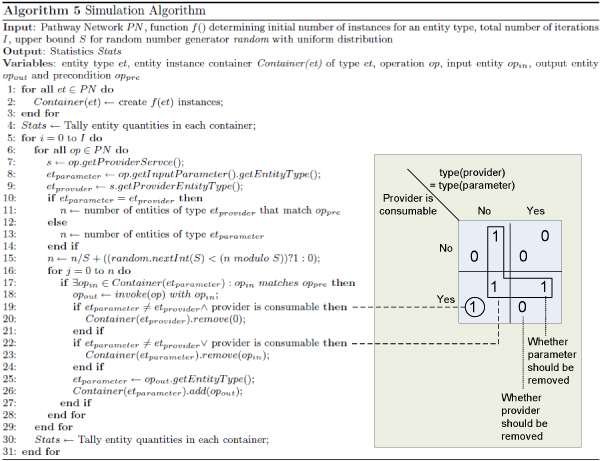
**Algorithm containing our simulation strategy**. Lower right corner depicts logic for removing entity instance after operation invocation. A value of 1 (or 0) indicates that the corresponding entity instance will (or will not) be removed after the operation invocation.

In Algorithm 5 (see Figure 12), an initial number of instances for each entity type *et* are first generated based on function *f(et)* (lines 01–03). It is conceivable that an expert may want to create different number of instances at the beginning for different entity types. Next, we conduct *I* iterations of operation invocations (lines 05–31). We take a snapshot of the quantities at the end of each iteration and before the very first iteration (lines 30 and 04). We determine the number of times the corresponding operation should be invoked based on the quantity of the corresponding service providing entity (lines 7 to 15). To make sure that an operation from a service providing entity of a small quantity also gets the chance to be invoked, a random number generator is used (line 15). Upon invocation of the operation, we remove corresponding entity instance based on the truth table depicted in the lower right corner of Figure 12. When we determine the provider should be removed (lines 19 to 21), we remove the first instance found in the corresponding container. Since the provider is not the input parameter, it is consequently not involved in the evaluation of the operation precondition. Thus we can remove any one instance found in the container. Lines 22 to 24 are for removing the input parameter instance when the corresponding condition is met. Finally, we add the output parameter instance to the corresponding entity container (lines 25 and 26).

## Results and discussion

**Figure 13:**
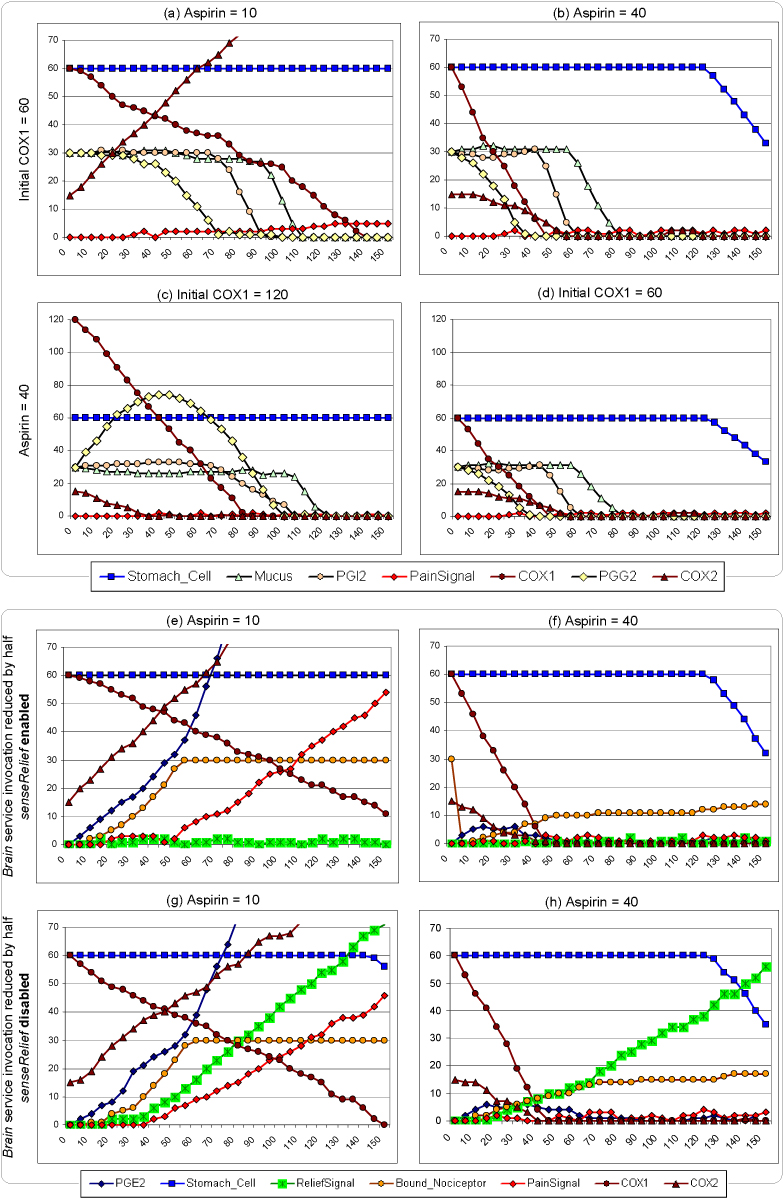
**Simulation Results**.

Figure 11 shows an example pathway network discovered using our mining algorithms. Simulation based on Algorithm 5 in Figure 12 is then conducted on the pathway network. Results obtained from each run by the PathExplorer are compiled into an Excel spreadsheet, which is then used to generate a plot such as those in Figure 13 (for the graph in Figure 11), where the horizontal axis is for the number of iterations and vertical axis is for quantity. Figure 13(a) shows that when the quantity of Aspirin is 10, there is no sign of stomach erosion. When the quantity of Aspirin increases to 40 in Figure 13(b), the quantity of stomach cell drops to around 30 after 150 iterations of operation invocation. This confirms the user hypothesis that Aspirin has a side effect on the stomach. In addition, we also noticed that given a fixed quantity of Aspirin, the reduction of the initial quantity of *COX1* also has a negative effect on the stomach (Figure 13(c) and 13(d)). When the initial quantity of *COX1* is high, it takes longer for all the *COX1* to get acetylated by Aspirin. As a result, enough *PGG2* and consequently *PGH2* and *PGI2* will be built up to feed into the *produceMucus* operation of the *StomachCellService*. As the initial quantity of *COX1* becomes smaller and while the depletion rate of Mucus by *GastricJuiceService* remains the same, less *Mucus* is being produced by the *StomachCellService* as less *PGI2* becomes available.

While Figures 9(a) to 9(d) clearly illustrate the relationships between Aspirin and Stomach_Cell, the relationship between the dosage amount of Aspirin and the sensation of pain is less obvious in these Figures. Except for Figure 13(a), which shows some accumulation of *PainSignal* when the quantity of Aspirin is 10, the rest of plots show no pattern of such accumulation or the variation thereof. A closer look at the highlighted pathway in Figure 11 reveals that this is actually consistent with the way the simulation is set up. Since *PainSignal* is created and then converted by the Brain to *ReliefSignal*, which disappears after it is sensed by *Nociceptor*, this whole path at the bottom actually acts as a 'leaky bucket'. To examine exactly what is going on along that path, we decided to make two changes in the simulation setting. First, we reduce the maximum frequency of invoking the Brain service to half that of *Nociceptor*. This creates a potential imbalance between the production rate of *PainSignal* and *ReliefSignal* since the processPain operation from the *BrainService* will be consequently invoked less frequently than the *sensePain* operation from the *NociceptorService*. Second, we disable the *senseRelief* operation of the *NociceptorService*. This essentially stops the leaking of the *ReliefSignal* that are generated as a result of the *PainSignal*. When we apply only the first change to the simulation, the imbalance of the processing rates for *PainSignal* and *ReliefSignal* results in a net accumulation of *PainSignal* when the quantity of Aspirin is 10 (Figure 13(e)). When the quantity is increased to 40 (Figure 13(f)), we see there are some occasional and temporary accumulation of *PainSignal*. Finally, we apply the second change along with the first one. Consequently, we notice that while the pattern of *PainSignal*'s accumulation hasn't changed much, there is a consistent accumulation of *ReliefSignal*. Since each *PainSignal* is eventually converted to a *ReliefSignal* by the Brain according to the highlighted pathway in Figure 11, the rate of *ReliefSignal*'s accumulation actually provides a much better picture on how fast *PainSignal* has been generated. We see that as the dosage amount of Aspirin increases, less *ReliefSignal* is generated, an indication that less *PainSignal* has been generated. Thus it is obvious that the increase of the dosage amount of Aspirin has a positive effect on the suppression of *PainSignal*'s generation. This confirms the other half of user's original hypothesis.

Simulation results such as these presented in Figure 13 provide useful information to a pathway analyst. They can be used to determine whether further more expensive *in vitro* and/or *in vivo* experiments are needed. If enough details are captured in the process models that the simulation is based on, then the simulation itself would present an inexpensive and accessible alternative to existing *in vitro* and/or *in vivo* exploratory mechanisms. Using the service-oriented simulation environment, the interrelationships among various entities involved in the pathway network can now be exposed in a more holistic fashion than traditional text-based pathway discovery mechanisms, which inherently lack the simulation capability.

## Conclusion

We proposed to model biological processes as Web service to bridge the gap between free-text description and traditional computer models of these processes. We presented our service mining tool named PathExplorer and demonstrated the feasibility of applying our service mining strategy to the discovery of pathways linking service models of biological processes. We described how PathExplorer identifies interesting segments in a pathway graph and automatically establishes a connected graph linking nodes that the user is interested in exploring. The graph, which is highlighted inside the discovered pathway network provides the user the basis for formulating hypothesis, which can then be tested out through simulation.

## List of abbreviations used

GraphML: (Graph Markup Language)
OWL-S: (Web Ontology Language based Web service ontology)
SOAP: (Simply Object Access Protocol)
WSDL: (Web Services Description Language)
WSDL-S: (WSDL with Semantics)
WSML: (Web Service Markup Language)
WSMT: (Web Service Modeling Toolkit)
WSMX: (Web Service Modeling eXecution environment)
XML: (eXtensible Markup Language).
